# MLST Genotypes and Antibiotic Resistance of *Campylobacter* spp. Isolated from Poultry in Grenada

**DOI:** 10.1155/2013/794643

**Published:** 2013-02-24

**Authors:** Diana Stone, Margaret Davis, Katherine Baker, Tom Besser, Rohini Roopnarine, Ravindra Sharma

**Affiliations:** ^1^School of Veterinary Medicine, St. George's University, P.O. Box 7, Grenada, West Indies; ^2^College of Veterinary Medicine, Washington State University, Pullman, WA 99164-7040, USA

## Abstract

This study determined whether multilocus sequence types (MLST) of *Campylobacter* from poultry in 2 farms in Grenada, West Indies, differed by farm, antimicrobial resistance and farm antibiotic use. Farm A used fluoroquinolones in the water and Farm B used tetracyclines. The E-test was used to determine resistance of isolates to seven antibiotics. PCR of the *IpxA* gene confirmed species and MLST was used to characterize 38 isolates. All isolates were either *C. jejuni* or *C. coli*. Farm antibiotic use directly correlated with antimicrobial resistance of *Campylobacter* isolates. Almost 80% of the isolates from Farm A were fluoroquinolone resistant and 17.9% of the isolates from Farm B were fluoroquinolone resistant. All *Campylobacter* isolates from Farm A were tetracycline sensitive, whereas 35.7% of isolates from Farm B were tetracycline resistant. Six previously recognized sequence types (STs) and 2 novel STs were identified. Previously recognized STs were those overwhelmingly reported from poultry and humans globally. Isolates with the same ST did not always have the same antibiotic resistance profile. There was little ST overlap between the farms suggesting that within-farm transmission of *Campylobacter* genotypes may dominate. MLST typing was useful for tracking *Campylobacter* spp. among poultry units and can help elucidate *Campylobacter* host-species population structure and its relevance to human health.

## 1. Introduction

Since the 1970s, *Campylobacter* has been recognized as one of the most common causative agents of bacterial foodborne illness [1, 2]. The genus *Campylobacter* consists of small Gram-negative, spirally curved rods and it is primarily the thermophilic species of *C. jejuni* and *C. coli.* that are responsible for human disease [3]. Infection in humans can be asymptomatic or present with a range of clinical symptoms from mild diarrhea to severe diarrhea with fever [4]. Complications may involve reactive arthritis [5] and Guillain-Barré syndrome [6]. The incidence of human *Campylobacter* infections is increasing worldwide, as well as the proportion of isolates resistant to fluoroquinolones, one of the primary classes of drugs used to treat human campylobacteriosis [7]. Animals are the natural reservoir hosts for *Campylobacter* species but the agent rarely causes disease in the animal host. The most common species of *Campylobacter* associated with human infections are *C. jejuni* and *C. coli* [8]. Both of these species have acquired fluoroquinolone resistance as well as resistance to other antibiotics [9–11]. It has been proposed that the poultry reservoir is the most important source of human infections and, in particular, the source for human fluoroquinolone-resistant *Campylobacter* isolates [1].

Grenada is a small tri-island state in the Eastern Caribbean. The country's livestock sector consists primarily of poultry, swine, and small ruminant production with poultry products having the highest demand in the local market. In 2009, Grenada produced over 1.5 million lbs of poultry, an increase over previous years. However, over 90% of meat requirements are met by imported products and an important national goal is to increase local poultry production by 60% in order to achieve a more self-sufficient market [12]. 

Information concerning the incidence of human campylobacteriosis in Grenada is not available, but World Health Organization report in 2005 from Grenada stated that gastroenteritis was an important cause of morbidity and mortality particularly among infants and children [13, 14]. More recently both *C. jejuni* and *C. coli* have been isolated from poultry in Grenada. Some isolates showed resistance to several antibiotics including fluoroquinolones, and importantly, antibiotic resistance profiles of isolates varied significantly among farms [15–17]. 

The objective of this study was to determine whether genetic clones of *Campylobacter* from poultry in Grenada differed by farm, antimicrobial resistance, and farm antibiotic use. To accomplish this multilocus sequence type (MLST), genotyping was performed on all *Campylobacter* isolates from two broiler farms which differed in their antibiotic use and in their prevalence of antibiotic resistant *Campylobacter*. MLST genotyping of *Campylobacter* is an important tool in elucidating the diversity of animal host *Campylobacter* isolates and provides not only information on transmission routes to humans but also on host-specific emergence of antibiotic resistant clones and their persistence within the animal host and the environment. The results of *Campylobacter* genetic population structures and antimicrobial resistance patterns within these two broiler farms in Grenada contribute to our understanding of the local and global epidemiology of antibiotic resistant poultry-associated *Campylobacter*. 

## 2. Material and Methods

### 2.1. Farm Factors

 Two poultry farms, designated Farm A and Farm B, participated in the study. A standardized questionnaire was completed at each farm by the owner/manager. Factors evaluated were housing type, flock size, feed composition and source, water source, presence of other domestic animals, use of footbaths, rodent control, husbandry practices, slaughter practices, flock health history, manure spread and disposal methods, and use of antimicrobial drugs [17]. A convenience sample of 25 6–8-week-old broilers were obtained from each farm between September 2009 and May 2011. Samples were collected at the farm immediately after the on-farm slaughter procedure. Cloacal swabs and postevisceration swabs of the abdominal cavity (visceral swabs) were obtained from each bird. Samples were transported on ice to St George's University School of Veterinary Medicine Diagnostic and Research Laboratory for processing in the same day. 

### 2.2. Bacterial Culture and Identification

Cloacal and visceral swabs were plated onto *Campylobacter* blood-free selective agar with cefoperazone and amphotericin B supplements (modified CCDA Preston plates) (Oxoid Ltd., Basingstoke, UK) and incubated under microaerophilic conditions at 42°C for 48 h using the GENbox microaer system (96126 BioMerieux UK Ltd., Basingstoke, UK). Presumptive *Campylobacter* colonies were identified by their typical grey-white, mucoid flat appearance on modified Preston CCDA plates. *Campylobacter* morphology was confirmed by Gram staining. Growths of pure cultures were transferred into 2% sterile skim milk in cryovials and stored at −85°C. To speciate the isolates, the hippurate test (Remel, Lennexa, KS, USA) was used as previously described [15]. Speciation was confirmed on DNA using a multiplex lpxA PCR that discerns between *C. jejuni*, *C. coli*, *C. lari*, and *C. upsaliensis*, based on amplicon size as previously described [18]. 

### 2.3. Antimicrobial Susceptibility Testing

Antimicrobial susceptibility/resistance of *Campylobacter* isolates was determined for the following seven antibiotics: gentamicin, chloramphenicol, ciprofloxacin, ampicillin, tetracycline, erythromycin, and metronidazole. The E-test (AB Biodisk Solna, Sweden) for minimum inhibitory concentration (MIC) was conducted according to the manufacturer's instructions on Mueller Hinton agar (Remel) as previously described [15]. A *C. jejuni* strain susceptible to all seven drugs and giving reproducible MICs was used as a control. The MIC of a drug was read directly from the scale printed on the E-test strip at the point of intersection between the zone of bacterial growth and the rest of the strip. Breakpoints established by the Clinical and Laboratory Standards Institute for the microbroth dilution test on aerobic bacteria were used to interpret MICs [19, 20]. MIC values used to classify a strain as resistant were as follows: ampicillin ≥32 *μ*g mL^−1^, chloramphenicol ≥32 *μ*g mL^−1^, ciprofloxacin ≥4 *μ*g mL^−1^, erythromycin ≥8 *μ*g mL^−1^, gentamicin ≥16 *μ*g mL^−1^, tetracycline ≥16 *μ*g mL^−1^ and metronidazole ≥16 *μ*g mL^−1^.

### 2.4. Multilocus Sequence Typing (MLST)

Stock cultures of the 51 isolates were grown on blood agar plates for 24 h at 42°C under microaerophilic conditions. Although most pathogenic species grow at 37°C, *C. jejuni* requires 42°C for optimum growth [21]. DNA was extracted using DNeasy Blood & Tissue Kits (Quagen Inc., Valencia, CA, USA) according to manufacturer's instructions, and samples were shipped to Washington State University College of Veterinary Medicine for MLST determination. Amplification of seven genes was carried out as described previously [22] using primer sequences obtained from http://pubmlst.org/campylobacter/info/primers.shtml. The amplification products were purified by precipitation with 20% polyethylene glycol 2.5 M NaCl and sequenced (Functional Biosciences Inc. Madison, WI, USA) using internal sequencing primers described on the same website. Each 20 *μ*L amplification reaction mixture contained Custom Genome Services (GCS, Pullman, WA, USA) master mix (14 *μ*L: 2 U taq polymerase, 0.2 mM dNTP's, 2 mM Mg), 1 *μ*M each primer, and 2 *μ*L gDNA template. PCR cycling conditions were as previously described by Schouls et al. [23], which is a modification of the procedure described by Dingle et al. [22].

The nucleotide sequences of the amplification products were determined at least once on each DNA strand using internal nested primers and BigDye Ready Reaction Mix (PE Biosystems, Foster City, CA, USA) in accordance with the manufacturer's instructions. Unincorporated dye terminators were removed by precipitation of the termination products with 95% ethanol. Sequence data were submitted to the *Campylobacter* PubMLST database for allele assignments.

### 2.5. Data Analysis

A two-tailed Fischer's exact test was used to compare the two farms for the number of broilers samples that were positive for *Campylobacter*, the number of *C. coli* and *C. jejuni* isolates, and the percent of isolates from each farm resistant to each of the seven antibiotics tested. A value of *P* ≤ 0.05 was considered significant. Sequence analysis and determination of ST and clonal complex were performed with the online-based MLST application for *Campylobacter* provided by SmartGene (Zug, Switzerland), which uses the PubMLST database http://pubmlst.org/campylobacter/ for ST designation.

 A neighbor-joining tree based on allelic sequences was constructed using the PHYLIP suite of programs available on the PubMLST web site http://pubmlst.org/analysis/. Tree drawing was performed using Phylodendron software also available at the same PubMLST site. Tree branches were labeled with corresponding clonal complex assignments.

## 3. Results

### 3.1. Farm Information

Both farms were located in the parish of St. Patrick and were separated by about two miles. At the time of sampling, Farm A housed approximately 8000 broilers and Farm B housed approximately 1000 broilers and 3000 layers. Only broilers were sampled for this study. Chickens from Farm A received the fluoroquinolone antibiotic, Norfloxacin, in their water. Chickens from Farm B received the tetracycline antibiotic, Oxytetracycline, in their water. Other than farm size, the type of antibiotic provided in the water and the fact that Farm B raised both broilers and layers, these farms did not differ in their management practices as determined by visual inspection of the premises and owner/manager responses to a questionnaire [17]. 

### 3.2. MLST and *Campylobacter* Strain Results

A total of 51 *Campylobacter* spp. isolates were obtained from broiler chickens on two farms in Grenada, Farm A (23 isolates; 20 from cloacal swabs and three from visceral swabs) and Farm B (28 isolates; 27 from cloacal swabs and one from a visceral swab). Based on this sample size, the farms did not differ significantly on the percent of sampled broilers positive for *Campylobacter* (88% Farm A and 96% Farm B, *P* = 0.6092) or on the percent of isolates that were identified as *C. coli* and *C. jejuni* by hippurate hydrolysis (8 *C. coli* and 15 *C. jejuni* isolates from Farm A and 13 *C. coli* and 15 *C. jejuni* isolates from Farm B, *P* = 0.5681). 

MLST genotyping was completed on 38 of the 51 isolates. For the other 13 isolates, ST determination was not possible because amplification products were not detected for one or more loci. These isolates represented both *C. jejuni* and *C. coli*. Six clonal complexes identified in this study have been previously described (STs-443, -828, -607, -353, -354, and -464). In addition, two novel STs were detected. One of the novel isolates, ST-5490, was assigned to the 353 clonal complex. The other novel isolate, ST-5491, has not yet been assigned to a clonal complex. One of the clonal complexes, ST-353, included two different STs, ST-353 and the novel ST-5490. Isolates from Farm A consisted of four previously reported STs (443, 353, 607, and 828) and two novel STs, ST- 5490 within the 353 clonal complex (two isolates) and ST-5491 (four isolates). Isolates from Farm B consisted of two PubMLST-reported STs (ST-354 and ST-464) and one novel ST, the ST-5491 (two isolates). This novel 5491 ST was the only MLST ST shared between the two farms. The two novel STs accounted for eight (21%) of the 38 typable isolates. These STs have been submitted to the PubMLST data base. Overall, the predominant ST was the ST-354 complex with 13 isolates (35%) and was only observed in *Campylobacter* isolates from Farm B (Table 1). The six previously reported clonal complexes detected in this study are associated with infections in chickens and humans (Table 2) and five of these are reported globally (Table 3).

 All isolates with confirmed STs were phenotypically typed by the hippurate test as either *C. jejuni* or *C. coli*, with 21 of the 38 isolates (55.3%) identified as *C. jejuni* and 11 (28.9%) of the isolates identified as *C. coli*. Among the *C. jejuni* and *C. coli* isolates typed in this study, six clonal complexes were identified for each, four of which included isolates of both species. Two of the clonal complexes included only *C. jejuni* isolates and two of the clonal complexes were observed only among *C. coli* isolates. The most frequent *C. jejuni* clonal complex was ST-354 (strain type 354) containing 11 of the 21 *C. jejuni* isolates. The seven *C. jejuni* STs identified in this study account for 19.4% of the *C. jejuni* human stool isolates since 2005 [24]. One of the two *C. coli* STs was the previously reported ST-828-1173 which contained five of the 11 *C. coli* isolates. This ST accounted for 74.6% of the *C. coli* human stool isolates reported to PubMLST between 2005 and 2011. Six of the 11 *C. coli* isolates were identified as the novel ST-5491, which was the only ST of this species observed on both farms.

**Figure 1:**
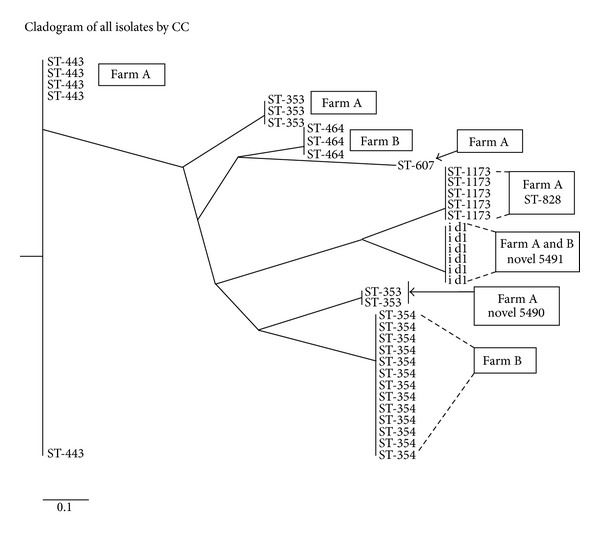
Neighbor-joining tree constructed from eight STs identified among 38 *Campylobacter jejuni/coli* isolates from broilers in Grenada at slaughter on two farms (A and B). Tree branches are labeled with the corresponding clonal complex assignments.

A cladogram constructed from the 38 *Campylobacter* MLST allelic profiles illustrates the clustering of the STs identified in this study. Some isolates from different farms occurred on branches that were closer than isolates within the same farm. For example, ST-464 clonal complex isolates from Farm B occupy the same branch in the tree as the ST-607 isolate from Farm A. Two clonal complex ST-353 isolates from Farm A (ST 5490) also branched off the same branch as clonal complex ST-354 isolates from Farm B (Figure 1).

### 3.3. Antibiotic Resistance

*Campylobacter* isolates from Farms A and B were significantly different with regard to resistance to gentamicin, ciprofloxacin, tetracycline, and metronidazole (Table 4). Seventeen of the 23 *Campylobacter* isolates from Farm A (73.9%) were resistant to ciprofloxacin whereas only five of the 28 isolates from Farm B (17.9%) were resistant to this antibiotic. None of the *Campylobacter* isolates from Farm A were resistant to tetracycline whereas 10 of the 28 isolates from Farm B (35.7%) were resistant to this class of antibiotic. Isolates from Farm B showed resistance to more classes of antibiotics and higher rates of resistance than isolates from Farm A. The farms did not differ significantly in the percent of isolates with multidrug resistance (30% of isolates from Farm A; 35.7% from Farm B). An analysis of all 51 *Campylobacter* isolates from these two farms showed that there was no significant difference between the percent of *C. jejuni* isolates resistant to at least one of the tested antibiotics (83%) and the percent of antibiotic resistant *C. coli* isolates (81%). Further, there was no significant difference between *C. jejuni* and *C. coli* isolates for the percent of resistance to ciprofloxacin (47% and 43%, resp.) or tetracycline (17% and 24%, resp.) (Fischer's exact two-tailed test, *P* > 0.05). 

All fluoroquinolone resistant isolates, regardless of MLST type, were from Farm A. All tetracycline resistant isolates, regardless of MLST type, were from Farm B. Multiple isolates of seven of the eight STs were obtained in this study. Isolates from only one ST for which multiple isolates were obtained (ST-353) showed a consistent antibiotic sensitivity/resistance profile, with all three isolates originating from Farm A and being resistant to fluoroquinolone and susceptible to tetracycline. Within each of the STs representing the remaining isolates, there was no consistent antibiotic resistance phenotype for fluoroquinolone or tetracycline. On Farm A, two of the four ST-443 isolates, four of the five ST-828 isolates, and one of the two ST-5490 isolates were resistant to fluoroquinolone whereas the others were sensitive. On Farm B, three of the 13 ST-354 isolates and one of the two ST-464 isolates were resistant to tetracycline whereas the others were sensitive. On Farm B, both of the novel 5491 isolates were sensitive to fluoroquinolone, but four of the isolates from Farm A were resistant to that antibiotic (Table 5).

 In one bird from Farm A, *Campylobacter* spp. were isolated from both the visceral and cloacal swabs for which complete MLST allelic profiles were obtained. The visceral swab isolate was assigned to ST-828 and was phenotypically identified as *C. coli*, whereas the cloacal swab isolate typed to ST-353 and was *C. jejuni*. Both isolates were resistant to fluoroquinolone and susceptible to tetracycline. Three of the visceral swab isolates from Farm B were typed to ST-354 and all were *C. jejuni*. Two of these isolates were obtained from broilers with MLST typable cloacal swab isolates. The ST of one of the cloacal swab isolates matched the ST of the visceral swab isolate from the same bird, ST-354. The visceral swab isolate was susceptible to tetracycline, whereas the cloacal swab isolate from this bird was resistant to tetracycline. Both isolates were susceptible to fluoroquinolone. The visceral and cloacal swab isolates from the other Farm B bird were both *C. jejuni* but did not match on either their MLST ST designation or their antibiotic susceptibility/resistance profiles. The MLST of the cloacal swab isolate was ST-464 and was resistant to tetracycline, whereas the MLST of the visceral swab isolate was ST-354 and was susceptible to tetracycline. Both isolates were susceptible to fluoroquinolone. 

## 4. Discussion

The aims of this study were to determine the prevalence, genetic diversity, and antibiotic resistance of *Campylobacter* isolates from broilers at slaughter on two farms in Grenada that differed in antimicrobial use. The high *Campylobacter* prevalence detected in broilers in this study is in agreement with previous findings in Grenada [15], Switzerland [25], France [26], Senegal [27], and Canada [28]. Also similar to those reports, the majority of isolates from this study were *C. jejuni*. In the earlier Grenada study, however, *C. coli* accounted for the majority of the isolates (60.9% of isolates compared to 32.8% for *C. jejuni*) which differed significantly with the results of the current study (*P* = 0.0154). The previous Grenada study [15] also identified *C. lari* isolates whereas it was not identified in broilers in this study. These differing results may reflect sampling time-line differences, different farms, farm facility or management differences, or sampling biases. Regardless of this, both *C. jejuni* and *C. coli* are prevalent in broilers in Grenada. 

In this study, eight STs were identified within six previously reported clonal complexes. Two of the STs identified were novel genotypes. One of these, ST-5491, was the only ST identified on both farms and may represent a local clone. The lack of farm MLST overlap suggests that farm-specific transmission pathways may predominate. However, a larger sample size at additional time points will be needed to assess this possibility. If farm-specific transmission of *Campylobacter* spp. does characterize Grenada poultry operations, it may reflect the current vertically integrated nature of poultry production where each farm controls all stages of production from day-old chicks through slaughter and processing. However, the cluster analysis (Figure 1) indicates that closely related STs were found on both farms suggesting possible interfarm transmission at some point.

Results of the MLST analysis suggest limited *Campylobacter* population diversity on Farm B with three STs and ST-354 accounting for over 72% of all isolates. In contrast, isolates from Farm A reflected six STs and no single ST accounted for more than 25% of the isolates. Predominant STs at a particular farm have been reported in several studies [29–31], and experimentally some strains are able to become dominant within a confined host-species population [32]. One study identified age of flock broilers as the most important factor correlated with increasing genetic diversity of *Campylobacter*, implying that genetic clones can accumulate within the flock and persist [33]. It is unlikely that the greater MLST diversity on Farm A compared to Farm B observed in this study reflects any difference in age of flock broilers or length of time these farms have been in production. Both of these farms were reestablished several months after being destroyed by Hurricane Ivan in 2004 and thus have been in continuous production for the same amount of time. Other factors that might contribute to differences in MLST diversity, such as antibiotic use and biosecurity measures prior to our study, or current proximity to other animal agriculture are unknown. A larger isolate set, however, might reveal a more diverse *Campylobacter* population on Farm B than what was identified in this study. 

 The major lineages identified in this study correspond to clonal complexes that are primarily associated with poultry and human disease (Table 2) and, except for ST-607, are geographically widely distributed (Table 3). Four of the six clonal complexes have been previously reported from Grenada [16, 24]. In general, results from this study support the previous observation that host association of *Campylobacter* genotypes transcends geographic variation [34]. It is of interest that Grenada is a tourist and medical/veterinary educational destination for people from the UK, the European community, and the US. This movement of humans and their pets to the island may impact the island's *Campylobacter* genotypic population structure.

In the study reported here, *C. coli* isolates represented a more restricted ST population structure than the *C. jejuni* isolates which is in agreement with a report from the UK [34]. Only one previously recognized *C. coli* clonal complex-ST was identified (ST-828-1173, and one novel ST (5491)). Of interest is that the previously reported *C. coli* ST-828 has been reported in several different countries and host species (Tables 2 and 3) and accounts for 74.6% of the *C. coli* human isolates reported to PubMLST. In a US study, *C. coli* isolates from both human cases and retail meats (beef, pork, and poultry) tended to cluster in the ST-828 complex with the majority of isolates showing ciprofloxacin, erythromycin, or multidrug resistance [35]. 

The fluoroquinolone and tetracycline resistance profiles of the 51 *Campylobacter* isolates showed a marked difference between Farm A (high ciprofloxacin resistance) and Farm B (high tetracycline resistance) that directly correlated with farm use of these antimicrobial classes. These results are compatible with antibiotic pressure contributing to both fluoroquinolone and tetracycline resistance, an association that has previously been described for both of these antibiotics [26, 36–39]. On Farm B, greater than 35% of the *Campylobacter* isolates were tetracycline resistant which is in agreement with several other studies on broilers [26, 28, 40–42]. Both *C. jejuni* and *C. coli* isolates from Farm A were resistant to ciprofloxacin, in agreement with other reports [26, 27, 41]. The percent of fluoroquinolone-resistant isolates on Farm A was high (73.9%), in agreement with other reports from farms where fluoroquinolones were used [41–45], and much higher than reports from countries where fluoroquinolones are banned or use is controlled [46]. Fluoroquinolone resistance on Farm A was also significantly higher than the 12.5% previously reported for poultry isolates from Grenada [15]. This difference may be due to the fact that data from the previous study included isolates from 10 farms and it is unlikely that fluoroquinolones were used on all farms. Isolates from the two farms in the present study also differed significantly for resistance to gentamicin and metronidazole, neither of which were used on these farms. Importantly, it is also recognized that fluoroquinolone-resistant *Campylobacter* spp. have been detected on farms that never used that class of antibiotic [47, 48]. Thus, factors in addition to farm use of antibiotics are likely contributing to antibiotic-resistant *Campylobacter* spp. in broilers in Grenada. 

Although antibiotic use did correlate with fluoroquinolone and tetracycline resistance, within each farm there was no association between MLST genotype and antibiotic resistance. This is similar to other reports for fluoroquinolone resistance [49, 50]. However, some studies report an association between specific MLST genotypes and resistance to fluoroquinolones [25, 51, 52], tetracycline [51, 52] or multidrug resistance which includes fluoroquinolones and tetracycline [53]. Where evaluated, STs associated with fluoroquinolone resistance contained mutations in the *gyrA* gene that is responsible for quinolone resistance, suggesting that either specific *Campylobacter* genotypes are prone to *gyrA* mutations or that clonal dissemination of resistant STs has occurred or that only a limited range of *gyrA* mutations confer fluoroquinolone resistance. Although *gyrA* mutations were not confirmed in the six STs on Farm A with fluoroquinolone resistance, results indicate that mutations in this gene may occur in a number of different STs on a single farm. 

Although further studies will be needed to determine a statistical association between antibiotic use and resistant *Campylobacter* strains, the presence of fluoroquinolone- and tetracycline-resistant *Campylobacter* isolates concurrent with farm use of these antibiotics in poultry production in Grenada indicates that there may be a need for more judicial use of antimicrobials on the island. Grenada is a small island state far from the US, UK, Europe, and Asia, and resistant *Campylobacter* strain emergence in Grenada could contribute to resistance globally. It has been noted that foreign travel is a risk factor for fluoroquinolone-resistant campylobacteriosis [54–56]. Furthermore, in popular European travel destinations, both fluoroquinolone and tetracycline resistance among poultry isolates is higher than in other locations [57]. Thus, the movement of people may play a role in transporting antibiotic-resistant strains to other locations. Tourism is the main industry in Grenada and protecting that economic resource, along with resident human health, is critical for this island state. 

In Grenada, poultry production is a vertically integrated, open air system of relatively small production units. Poultry production is often in close association with back yard or small-scale sheep, goat, and swine production units. Thus, there is ample opportunity for movement of *Campylobacter* spp. among these host species. This type of animal agriculture is common in many parts of the world and findings from Grenada may shed light on *Campylobacter* population structure, spread, and resistance relevant to many geographic areas. Data from studies of *Campylobacter* in sheep, goats, and pigs across all parishes in Grenada show low shedding rates in sheep and goats (3-4%, unpublished data) and high rates in pigs (72%) [58]. Antimicrobial and MLST analysis will determine whether any of these mammalian isolates correspond to isolates identified in poultry. This, along with genetic and antimicrobial resistance analysis of human case isolates, will give a more comprehensive picture of *Campylobacter* host-species population structure and its relevance to human health in Grenada and to campylobacteriosis in countries with similar food animal production systems.

## Figures and Tables

**Table 1:** Clonal complexes and allelic profiles from 38 *Campylobacter jejuni/coli* strains isolated from broilers in Grenada.

Farm	CC	ST	Allele number						
			ASP	GLN	GLT	GLY	PGM	TKT	UNC
A	ST-443	51 (5)	7	17	2	15	23	3	12
	ST-828*	1173 (5)	33	39	30	82	113	56	17
	ST-607	2927 (1)	166	2	5	10	151	3	1
	ST-353	353 (3)	7	17	5	2	10	3	6
		**5490 (2)** ^†^	7	84	5	2	11	1	6
	‡	**5491 (4)** ^†^	129	39	65	82	113	47	17

B	ST-354	354 (13)	8	10	2	2	11	12	6
	ST-464	464 (3)	24	2	2	2	10	3	1
	‡	**5491 (2)** ^†^	129	39	65	82	113	47	17

Numbers in parentheses after each ST denote the number of isolates.

CC: clonal complex.

ST: sequence types.

*Clonal complex associated with *C. coli*.

^†^Novel STs identified.

^‡^No clonal complex assigned yet.

**Table 2:** The distribution of source host species for clonal complexes detected in Grenada *Campylobacter jejuni/coli* broiler isolates represented in the international PubMLST database.

Source*	ST-353 clonal complex *N* (%)	ST-354 clonal complex *N* (%)	ST-443 clonal complex *N* (%)	ST-464 clonal complex *N* (%)	ST-607 clonal complex *N* (%)	ST-828 clonal complex *N* (%)
Ruminant	2 (0.5)	10 (4.2)	2 (0.9)	0 (0)	1 (0.8)	162 (10.4)
Chicken	92 (22.8)	64 (27.1)	60 (27.4)	24 (24)	35 (29.7)	523 (33.4)
Other poultry	7 (1.7)	3 (1.3)	1 (0.5)	1 (1)	0 (0)	96 (6.1)
Human	301 (74.7)	158 (66.9)	156 (71.2)	75 (75)	82 (69.5)	378 (24.2)
Pig	1 (0.2)	1 (0.4)	0 (0)	0 (0)	0 (0)	405 (25.9)

Total	403 (100.0)	236 (100.0)	219 (100.0)	100 (100.0)	118 (100.0)	1564 (100.0)

*Ruminant: calf, cattle, cow's milk, goat, sheep; Chicken: chicken, chicken offal or meat; Other poultry: goose, turkey; Human: human stool, human blood, human unspecified; Pig: pig, pork offal or meat.

**Table 3:** *Campylobacter *clonal complex STs identified in broilers from farms A and B that have been previously reported to PubMLST from Grenada and other countries.

	ST-353	ST-354	ST-443	ST-464	ST-607	ST-828
	353	354	51	464	2927	1173
UK	x	x	x	x		x
The Netherlands	x	x	x	x		
Germany	x	x	x	x		
Spain				x		x
Switzerland	x			x		
Greece	x	x				
Belgium		x				
USA	x	x	x			x
Canada	x	x	x	x		
Australia		x	x			
Thailand		x	x	x	x	
Japan				x		
Curacao	X		x			
Grenada	X	x	X			x
Number of records in PubMLST	50	50	97	30	2	6
Species	*C. jejuni *	*C. jejuni *	*C. jejuni *	*C. jejuni *	*C. jejuni *	*C. coli *

**Table 4:** Antibiotic sensitivity/resistance profiles of 51 *Campylobacter jejuni/coli* isolates from broilers on Farms A and B in Grenada.

Antibiotic*	Farm A	Farm B	*P* value comparing
	Sen/Resist (% R)	Sen/Resist (%R)	% resistant^†^
Gen	23/0 (0%)	22/6 (21.4%)	***P = 0.0265 ***
Chlor	22/1 (0.04%)	25/3 (10.7%)	*P* = 0.6173
Cipro^‡^	6/17 (73.9%)	23/5 (17.9%)	***P = 0.0001 ***
Amp	20/3 (13%)	27/1 (3.6%)	*P* = 0.3158
Tetra^$^	23/0 (0%)	18/10 (35.7%)	***P = 0.0011 ***
Eryth	23/0 (0%)	23/5 (17.9)	*P* = 0.0562
Metroni	17/6 (26.1%)	7/21 (75.0%)	***P = 0.0007 ***

*Gen: gentamicin; Chlor: chloramphenicol; Cipro: ciprofloxacin; Amp: ampicillin; Tetra: tetracycline; Eryth: erythromycin; Metroni: metronidazole.

^†^Fischer's exact two-tailed test.

^‡^Antibiotic used on Farm A.

^$^Antibiotic used on Farm B.

**Table 5:** Fluoroquinolone and tetracycline sensitivity/resistance of *Campylobacter jejuni/coli* isolates by MLST profile from broilers on Farms A and B in Grenada.

Farm/antimicrobial used	CC	ST	Antimicrobial sensitivity/resistance	
			Fluoroquinolone	Tetracycline
Farm A-fluoroquinolone	ST-443	51 (3)	S	S
		51 (2)	**R**	S
	ST-828	1173 (1)	S	S
		1173 (4)	**R**	S
	ST-607	2927 (1)	**R**	S
	ST-353	353 (3)	**R**	S
		5490 (1)	S	S
		5490 (1)	**R**	S
	No CC assigned	5491 (4)	**R**	S

Farm B-tetracycline	ST-354	354 (10)	S	S
		354 (3)	S	**R**
	ST-464	464 (2)	S	S
		464 (1)	S	**R**
	No CC assigned	5491 (2)	S	S

CC: clonal complex.

ST: sequence types.

Numbers in parentheses after each ST denote the number of isolates.

S: sensitive.

R: resistant.
